# The Byways of Infection

**Published:** 1901-10-12

**Authors:** 


### THE BYWAYS OF INFECTION.

We seem to be as far as ever from coming to the end of the byways of infection. As is well known, the typhoid bacillus is poured out in large quantities in the urine of patients suffering from, and often for some time after recovery from, typhoid fever, and the necessity for disinfecting the urine as well as the stools of patients affected with that disease has lately been strongly insisted on. Now it seems that the same is true of tuberculosis. According to Dr. Foulerton and Mr. Hillier,[1] whose conclusions are confirmed by Dr. Hugh Walsham,[2] the urine of patients suffering from tuberculosis is liable to contain tubercle bacilli, although only in small numbers. Of 18 patients suffering from more or less chronic tuberculous pulmonary phthisis, the urine contained B. tuberculosis in nine. In six of these nine cases the absence of tuberculosis of the urinary tract was proved by post-mortem examination, and there was no reason to suspect that the condition existed in any of the other three. So far as it is permissible, then, to generalise from a small number of cases, it may be said that the urine in 50 per cent. of fairly advanced cases of tuberculous pulmonary phthisis contains B. tuberculosis in a condition which is virulent to the guinea-pig. It is to be noted, however, that, judging from the mode of reaction manifested by the guinea-pigs which were made the subject of experiment, the bacilli existed in only very small numbers in the urines examined. Still, the point remains that in urine secreted by the apparently healthy kidneys of tuberculous patients the bacilli existed—another byway of infection.

[1] Brit. Med. Journ., Sept. 21. [2] Brit. Med. Journ., Sept. 28.

